# Mmp1 Processing of the PDF Neuropeptide Regulates Circadian Structural Plasticity of Pacemaker Neurons

**DOI:** 10.1371/journal.pgen.1004700

**Published:** 2014-10-30

**Authors:** Ana Depetris-Chauvin, Ágata Fernández-Gamba, E. Axel Gorostiza, Anastasia Herrero, Eduardo M. Castaño, M. Fernanda Ceriani

**Affiliations:** 1 Laboratorio de Genética del Comportamiento, Fundación Instituto Leloir and Instituto de Investigaciones Bioquímicas-Buenos Aires (IIB-BA, CONICET), Buenos Aires, Argentina; 2 Laboratorio de Amiloidosis y Neurodegeneración, Fundación Instituto Leloir, IIB-BA-CONICET, Buenos Aires, Argentina; Washington University Medical School, United States of America

## Abstract

In the *Drosophila* brain, the neuropeptide PIGMENT DISPERSING FACTOR (PDF) is expressed in the small and large Lateral ventral neurons (LNvs) and regulates circadian locomotor behavior. Interestingly, PDF immunoreactivity at the dorsal terminals changes across the day as synaptic contacts do as a result of a remarkable remodeling of sLNv projections. Despite the relevance of this phenomenon to circuit plasticity and behavior, the underlying mechanisms remain poorly understood. In this work we provide evidence that PDF along with matrix metalloproteinases (Mmp1 and 2) are key in the control of circadian structural remodeling. Adult-specific downregulation of PDF levels *per se* hampers circadian axonal remodeling, as it does altering Mmp1 or Mmp2 levels within PDF neurons post-developmentally. However, only Mmp1 affects PDF immunoreactivity at the dorsal terminals and exerts a clear effect on overt behavior. *In vitro* analysis demonstrated that PDF is hydrolyzed by Mmp1, thereby suggesting that Mmp1 could directly terminate its biological activity. These data demonstrate that Mmp1 modulates PDF processing, which leads to daily structural remodeling and circadian behavior.

## Author Summary

Circadian clocks have evolved as mechanisms that allow organisms to adapt to the day/night cyclical changes, a direct consequence of the rotation of the Earth. In the last two decades, and due to its amazing repertoire of genetic tools, *Drosophila* has been at the leading front in the discovery of genes that account for how the clock operates at a single cell level, which are conserved throughout the animal kingdom. Although the biochemical components underlying these molecular clocks have been characterized in certain detail, the mechanisms used by clock neurons to convey information to downstream pathways controlling behavior remain elusive. In the fruit fly, a subset of circadian neurons called the small ventral lateral neurons (sLNvs) are capable of synchronizing other clock cells relying on a neuropeptide named pigment dispersing factor (PDF). In addition, a number of years ago we described another mechanism as a possible candidate for contributing to the transmission of information downstream of the sLNvs, involving adult-specific remodeling of the axonal terminals of these circadian neurons. In this manuscript we describe some of the molecular events that lead to this striking form of structural plasticity on a daily basis.

## Introduction

The rotation of the earth around its own axis imposes cyclic changes on environmental conditions, primarily through variations on luminosity and temperature. The existence of an endogenous, self-sustained and entrainable circadian clock in almost every living organism allows them to anticipate those daily changes and concomitantly adapt their physiology and behavior to a changing environment [1]. Although the biological processes that present circadian modulation may differ depending on the ecological niche that each species occupies, the molecular basis of the circadian clock shows an intriguing similarity through evolution. Briefly, circadian clocks depend on the coordinated activity of transcriptional/translational feedback loops of clock genes running within specific pacemaker cells [2]. In *Drosophila melanogaster* this molecular clock is allocated in a circadian network of approximately 150 neurons in the adult brain, and the coordinated activity of the whole circuit is necessary for plastic responses to different environmental stimuli (revised in [3]). However, under constant conditions, circadian locomotor activity strongly depends on the activity of 8 neurons located on the accessory medulla on each side of the adult brain [4], [5], which are known as the small and large lateral ventral neurons (sLNvs and lLNvs, respectively); all of them express the PIGMENT DISPERSING FACTOR neuropeptide and are therefore also known as PDF neurons. Several experiments have determined that the sLNvs are in fact in charge of determining the endogenous period of locomotion under constant conditions [6], [7] while the lLNvs appear to be involved in sleep and arousal [8]–[10]. How the circadian network transmits time of day information is still under debate but the activity of the PDF neuropeptide [5], [11] and, more specifically, daily changes on immunoreactivity of the PDF-containing dense core vesicles at the axonal terminals [12] as well as circadian changes on electrical activity [13] have been proposed as putative mechanisms. In addition, we have demonstrated that the sLNvs axonal terminals exhibit a higher degree of complexity during the day and a reduced complexity during the night accompanying the daily changes in PDF levels [14]. Interestingly, this circadian structural plasticity may result in a change in synaptic partners at different times of the day and might offer another relevant mechanism to transmit time of day information [15].

Axonal structural plasticity related to circuit assembly during development has extensively been studied but only recently its occurrence during adulthood in the absence of physical lesions has been reported [16], [17]. Axonal remodeling during adulthood is recruited to adjust biological processes such as axonal injury, adult neurogenesis, sensory experience, learning and memory [18] and as a response to homeostatic regulation followed by sleep deprivation [19], [20]. In addition to such homeostatic changes, endogenous mechanisms determine circadian axonal remodeling of peripheral circuits [21], [22] and, also, of central neurons relevant to circadian rhythms [14], [19], [23]. The molecular and cellular processes underlying such axonal plasticity during adulthood are not clear, but different mechanisms might be engaged in the remodeling of specific neurons [18]. In the case of circadian structural plasticity, it is expected that at least part of the molecules responsible for orchestrating changes in axonal terminals show circadian modulation of gene expression, protein stability and/or activity. In this regard, we found matrix metalloproteinases (Mmps) to be attractive candidates to modulate circadian axonal remodeling of PDF neurons.

In *Drosophila* there are only two Mmps, Mmp1 and Mmp2, and their action is involved in several processes ranging from tissue remodeling [24], tumor invasiveness [25], axon guidance, axonal fasciculation [26] and dendritic remodeling [27]. Interestingly, cell-type specific gene-expression profiling revealed enrichment of Mmp1 and 2 expression in sLNv neurons at the beginning of the night [28]. Moreover, Mmp1 appears to be a direct target of CLOCK, a central component of the molecular clock [29].

In this study we investigated the molecular mechanisms underlying circadian structural remodeling of PDF axonal terminals. We demonstrated that both Mmps are key players in the remodeling of PDF neurons, promoting a reduction of the complexity of the axonal arborizations. In concert with the action of Mmps, fine tuning of the dorsal arborizations also depends on the PDF neuropeptide. Furthermore, we found that cell-type autonomous modulation of Mmp1 levels, unlike Mmp2, regulates the levels of the PDF neuropeptide, highlighting the relevance of Mmp1 in the determination of the neuronal output of the central pacemaker cells.

## Results

### Matrix metalloproteinases are key players of the structural plasticity of PDF neurons

**Figure 1:**
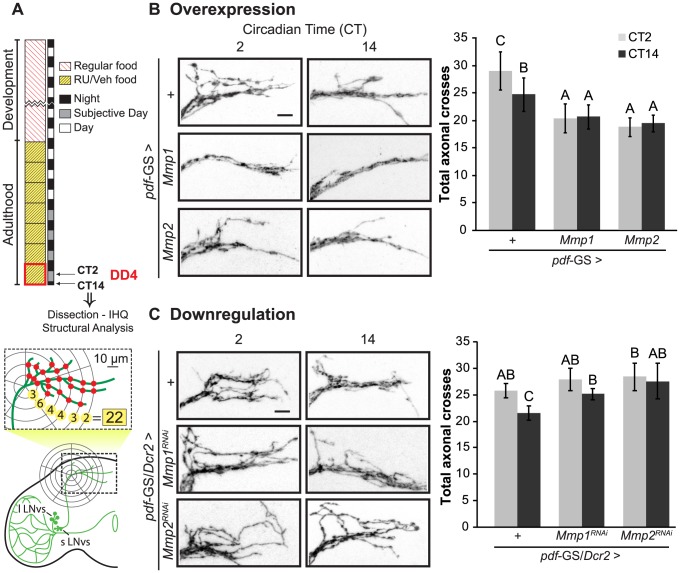
Mmps are key players of the structural plasticity of PDF neurons. **A.** Schematic diagram illustrating the standard protocol and method for the analysis of the complexity of the PDF axonal arbor on confocal images. In all figures “VEH” and “RU” stand for “vehicle”- and “RU486”-containing fly food. **B.** Adult-specific Mmp overexpression triggers structural phenotypes. **Left panel.** Representative confocal images of GFP immunoreactivity at the dorsal protocerebrum at the early subjective day (CT2) and early subjective night (CT14) during the 4^th^ day of constant darkness (DD4). **Right panel.** Quantitation of total axonal crosses. Wild type flies display circadian structural remodeling of axonal terminals while animals overexpressing Mmp1 or Mmp2 show reduced and constant complexity. Throughout the manuscript all experimental groups include CD8GFP, so the control group “+” refers to a single copy of *CD8GFP;pdf*-GS. Throughout the manuscript the average ± standard error of the mean is shown. **C.** Adult-specific Mmp downregulation also affects dorsal axonal branches. Silencing either Mmp1 or Mmp2 abolished circadian structural plasticity leading to a more complex structure clamped at the daytime configuration. Data represents the average of 4 to 5 experiments and a minimum of 27 brains were analyzed per CT/Genotype. Different letters indicate statistically significant differences with a p<0.05 (Two-way ANOVA with a Duncan *post-hoc* test). For more details, see the Statistics section in Materials and Methods. “+” refers to a single copy of the *pdf*-GS/*CD8GFP*;*Dcr2* transgenes. In both experiments all the experimental groups include RU to induce expression. Scale: 10 µm.

To examine a possible contribution of Mmps to the circadian structural plasticity of the sLNvs axonal terminals we altered Mmp1 or Mmp2 expression specifically in PDF neurons and analyzed the degree of arborization at the dorsal protocerebrum at two time points during the subjective day, at Circadian Time 2 (CT2, 2 hours after the lights should have been on) and CT14 (2 hours after lights should have been off) (**Figure 1A**). We restricted our treatment to the adult stage by using the *pdf*-GS RU486-inducible GeneSwitch strain recently described [30] to bypass any potential developmental effect. As previously described [14], control flies displayed a more complex arborization pattern during the early subjective day (CT2) and less arborized display during the early subjective night (CT14). On the contrary, adult-specific Mmp1 or Mmp2 overexpression in PDF neurons abolished any remodeling of dorsal projections, leading to a non-oscillating and less complex circuit that shows even fewer axonal crosses than the nighttime control neurons (**Figure 1B**). Overexpression with independent transgenic lines rendered similar results (**Figure S1 A**). A more detailed analysis of structural complexity indicated that Mmp1 does not affect its total axonal length while it does reduce the complexity of the arborizations all along the axonal projections. In contrast, Mmp2 has a significant effect on total axonal length indicating that the changes trigged by Mmp2 overexpression involve modification of the length of axonal terminals (**Figure S1 B–D**). Thus, although both Mmps impact the circadian remodeling of PDF neurons, the underlying mechanisms are not necessarily the same.

To corroborate Mmp1 presence in PDF neurons, immunohistochemistry on whole mount brains was carried out during both transitions, dark to light and light to dark. Despite its overall low levels (that precluded reliable detection in the sLNvs), Mmp1 was more frequently detected in the large LNvs somas at dusk rather than at dawn (Figure S1F), which is in agreement with the transcription profile reported for this gene (roughly undetectable at ZT0 and detectable at ZT12, [31]).

We extended our analysis on the role of Mmps in circadian plasticity through RNAi-mediated downregulation of Mmp1 or Mmp2 expression. Co-expression of Dicer2 ensured a drastic reduction of Mmp1 and Mmp2 levels since expression on the whole animal through the constitutive promoter *actin*-GAL4 caused larval or pupal lethality as it is the case for null mutants ([24]). The acute activation of a component of the silencing machinery did not affect circadian remodeling *per se*, since control flies overexpressing Dicer2 showed changes in the degree of complexity reminiscent of wild type animals (Compare “+” in Figures 1B and 1C). Adult specific downregulation of Mmp1 or Mmp2 disrupts the daily changes in the complexity, although the structure is fixed on a daytime configuration comparable to the one of control animals (**Figure 1C**). Importantly, independent RNAi lines triggered similar effects (**Figure S1G**). Downregulation of Mmp2 but not Mmp1 significantly increased the length of the main axonal branches, underscoring that they affect the structure of PDF neurons through different mechanisms (**Figure S1 E**).

In conclusion both Mmps are key players in the circadian modulation of the fine structure of the sLNvs, where high Mmp levels promote a less complex arborization, as the one observed during the early night, while low Mmp levels lead to the opposite effect.

### Mmp1 modulates behavioral rhythmicity

**Figure 2:**
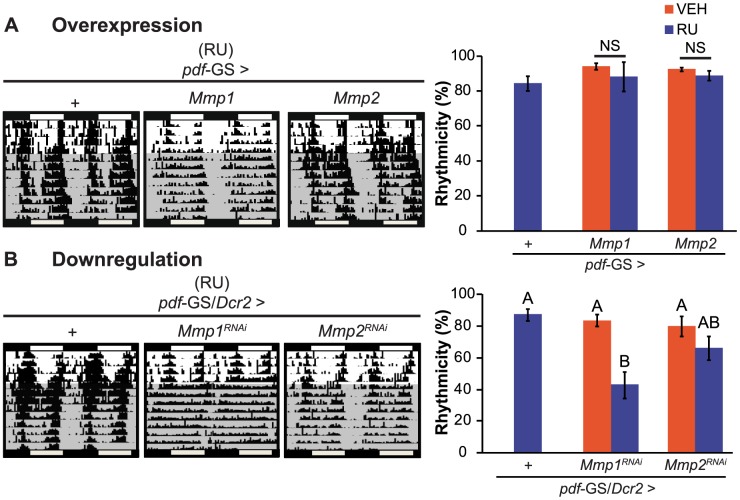
Mmp1 modulates behavioral rhythmicity. **A.** Representative actograms (**left panel**) and quantitation of percentage of rhythmicity (**right panel**) from overexpression experiments. Locomotor activity of individual flies was recorded for 4 days under light-dark cycles and then transferred to constant darkness (gray area) for 9 additional days. Overexpression of Mmp1 or Mmp2 with one UAS copy does not affect circadian locomotor activity. “+” in the x axis refers to a single copy of *CD8GFP*; *pdf*-GS. NS, non significant. **B.** Adult-specific Mmp downregulation trigger opposite effects on locomotor rhythmicity. Silencing Mmp1 but not Mmp2 alters normal circadian locomotor activity. “+” in the x axis refers to a single copy of *CD8GFP*; *pdf*-GS. Data represents at least 3 independent experiments and a minimum of 32 flies per Genotype/Condition were analyzed. Different letters indicate statistically significant differences with a p<0.05 (Two-way ANOVA with a Duncan *post-hoc* test). For other controls and measurements of endogenous period see Table S1.

To examine if structural plasticity of PDF neurons is necessary for the control of behavioral rhythmicity we sought to determine if flies that do not present cyclic axonal remodeling show any disruption on circadian locomotor activity. Control flies and those overexpressing either Mmp1, Mmp2 or specific RNAi constructs directed to Mmp1 or Mmp2 were recorded for their locomotor activity during 4 days in the presence of external cues (cycles of 12 hours of lights and 12 hours of darkness, LD) and then released to constant darkness (DD) to evidence the circadian control of behavior. Wild type flies present a clear rhythm in their locomotor activity both in the presence of synchronizing cues (LD) and in constant conditions (DD). In DD, this rhythm has a period of approximately 24 h and flies consolidate their activity along the subjective day. In this experiment, genetic as well as the non-induced controls (flies including all transgenes kept in the absence of the chemical inducer) behave as wild type animals with largely rhythmic individuals with an endogenous period close to 24 h (**Figure 2** **and Table S1**). Overexpression of Mmp1 or Mmp2 with a single copy of the transgenes did not cause any significant effect on locomotor rhythmicity (**Figure 2A**) although increasing Mmp1 levels through the addition of a second UAS-transgene produced a significant reduction of behavioral rhythmicity (**Figure S2**). Interestingly, downregulation of Mmp1 but not Mmp2 led to a severe deconsolidation of locomotor activity that resulted in a clear reduction in the rhythmicity of the population (**Figure 2B**). Those that remained rhythmic displayed an endogenous period indistinguishable from control flies (**Table S1**), highlighting a specific effect of Mmp1 on the consolidation of rhythmic locomotor activity as opposed to period determination.

### Mmp1 acts in concert with Fasciclin 2 and Ecdysone Receptor

Given the complexity and extent of daily reorganization we reasoned that other molecules might be implicated in fine tuning the structure of PDF neurons along the day. The analysis of structural changes in the same brain over time indicates that axonal projections of sLNvs endure changes in pruning and neuritogenesis as well as changes in the degree of fasciculation [15]. Consistent with such contribution, it has recently been shown that Fasciclin 2 (Fas2), the ortholog of mammalian NCAMs in *Drosophila*, plays a role in the structural remodeling of sLNv axonal projections [32]. In addition, Mmps act in concert with Fas2 promoting the fasciculation of axonal bundles during the development of neuronal circuits [26] but also interact with the Ecdysone pathway assisting dendritic pruning [27]. Taking this information into account, we sought to examine whether these two programs were also recruited in PDF neurons to accomplish their circadian structural remodeling. To shed light on this possibility, we tested if modulating Fas2 or Ecdysone Receptor (EcR) levels could modulate the structural defects caused by high Mmp1 levels. RNAi-mediated downregulation of Fas2 levels in the context of Mmp1 overexpression partially restored the complexity of axonal arborizations (**Figure S3 A**). Noteworthy, this rescue was not a byproduct of the inclusion of additional UAS constructs since additional transgenes in the context of Mmp1 overexpression did not alter its phenotype (**see below**). On the other hand, expression of a RNAi line directed to EcR in PDF neurons rescued the structural plasticity to wild type levels, antagonizing the effects caused by Mmp1 overexpression. Along these lines, downregulation of EcR affected PDF neurons *per se*, clamping the structure in the more complex, highly arborized, configuration (**Figure S3 B** and [33]).

Together these results demonstrate that the daily axonal remodeling of PDF neurons is a complex and highly regulated process that depends on the concerted activity of Mmps, Fasciclin 2 and the Ecdysone Receptor.

### Mmp1 expression in PDF neurons affects PDF levels

**Figure 3:**
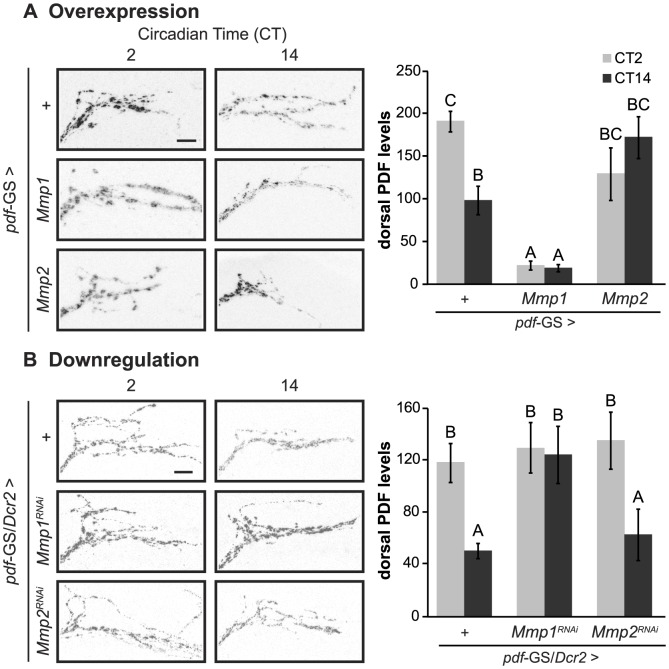
Cell autonomous Mmp1 expression regulates PDF levels. **A.** Overexpression experiments **Left panel.** Representative confocal images of PDF immunoreactivity at the dorsal protocerebrum taken during CT2 and CT14 on DD4. **Right panel.** PDF levels at the dorsal protocerebrum. Control flies exhibit circadian oscillation of PDF levels, while Mmp1 overexpression reduces PDF to levels lower than those observed at nighttime in controls. In contrast, Mmp2 overexpression leads to intermediate levels. “+” in the x axis refers to a single copy of *CD8GFP*; *pdf*-GS. **B.** Downregulation experiments. Reducing Mmp1 but not Mmp2 levels abolishes circadian oscillations in PDF immunoreactivity to constant daytime levels. “+” in the x axis refers to a single copy of *CD8GFP*, *Dcr2*; *pdf*-GS. Data represents the average (± standard error of the mean) of at least 3 independent experiments and a minimum of 23 flies per Genotype/CT were analyzed. Different letters indicate statistically significant differences with a p<0.05 (Two-way ANOVA with a Duncan *post-hoc* test). In overexpression experiments logarithmic transformation was applied to fulfill ANOVA requirements. In both experiments all the experimental groups include RU to induce expression. Scale: 10 µm.

PDF is crucial for the proper control of circadian locomotor activity since *pdf^o1^* and *pdf* Receptor (*pdfR/han*) mutants largely become arrhythmic under DD conditions [5], [34],[35]. Therefore, we wondered if the behavioral phenotypes described for flies with Mmp1 missexpression were reflecting an alteration of PDF signaling. To address this possibility we measured the levels of the neuropeptide at the dorsal protocerebrum by immunohistochemistry during the early subjective day (CT2) and night (CT14). In control animals, PDF immunoreactivity changes at the dorsal terminals, with high levels at CT2 and low levels at CT14 (**Figure 3 A–B** **, upper panels**). Overexpression of Mmp1 or Mmp2 affected PDF immunoreactivity and disrupted its circadian oscillation. Mmp1 effect was far more severe, resulting in reduced PDF levels at both timepoints to an extent that reached statistical significance; on the contrary, Mmp2 affected PDF levels rather subtly and led to intermediate levels that did not significantly differ from any timepoint in control flies (**Figure 3A**). Overexpression with independent transgenic lines retrieved similar results (**Figure S4 A**). RNAi analysis showed that reduced Mmp1 but not Mmp2 levels abolished the circadian oscillation in PDF immunoreactivity, resulting in levels reminiscent of the daytime configuration (**Figure 3B**). Interestingly, the fact that downregulation of Mmp1 but not Mmp2 affects PDF immunoreactivity correlates with the specific effect of silencing Mmp1 on locomotor activity, suggesting that clamping PDF at high levels might be the cause of the behavioral phenotypes observed.

### The neuropeptide PDF directs the remodeling of PDF axonal processes

**Figure 4:**
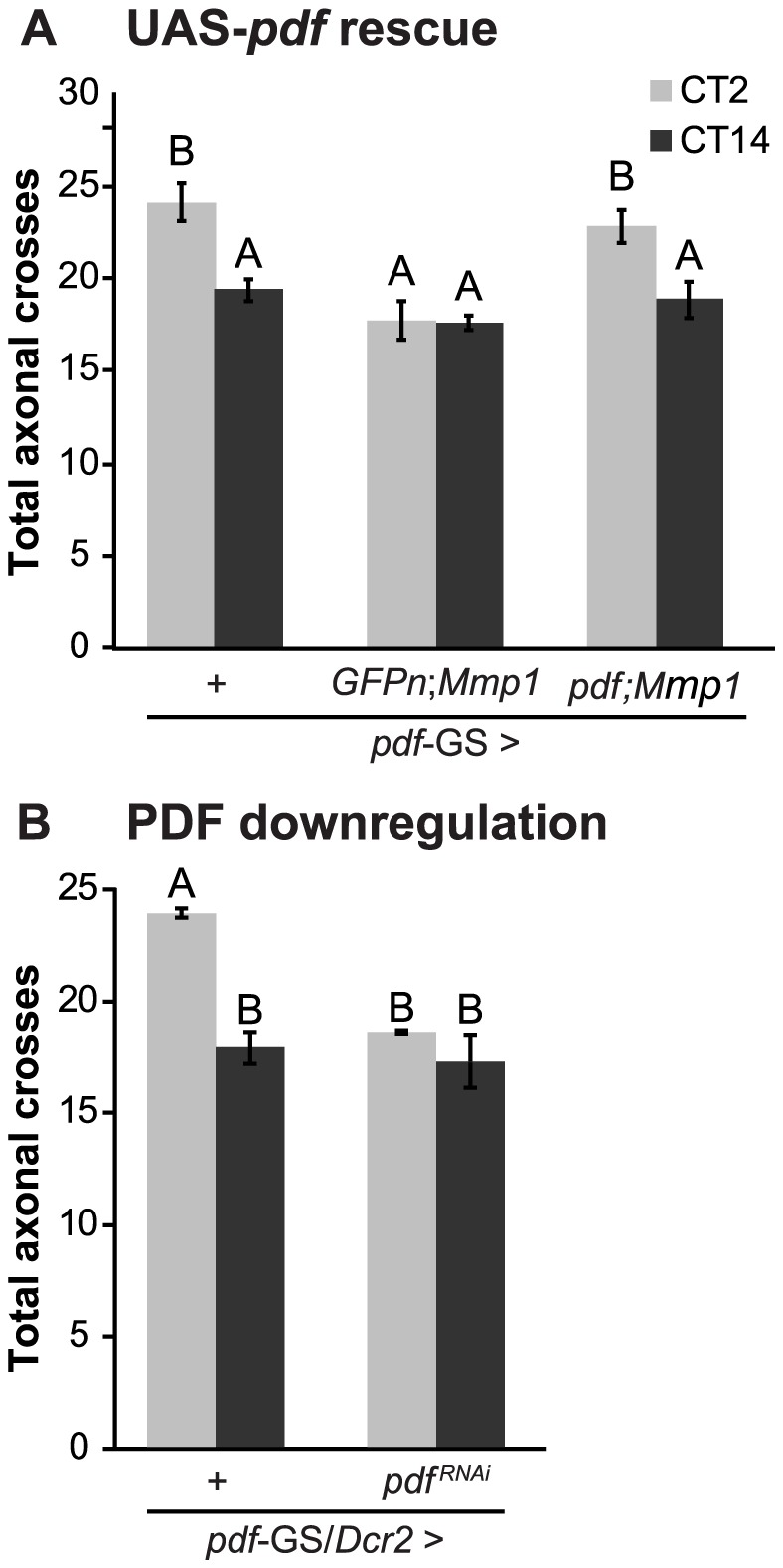
PDF defines the axonal remodeling of its own neurons. **A.** Quantitation of total axonal crosses from UAS-PDF rescue experiments. Overexpression of PDF rescues the structural plasticity defects caused by Mmp1 overexpression. “+” in the x axis refers to a single copy of *CD8GFP*; *pdf*-GS. Data represents the average (± standard error of the mean) between 3–5 independent experiments and a minimum of 21 flies were analyzed per Genotype/CT. **B.** PDF downregulation prevents circadian axonal remodeling of sLNv terminals and reduces daytime complexity to nighttime levels. “+” in the x axis refers to a single copy of *CD8GFP*, *Dcr2*; *pdf*-GS. Data represents the average (± standard error of the mean) between 3 independent experiments and a minimum of 25 flies were analyzed per Genotype/CT. In both experiments different letters indicate statistical differences with a p<0.05 (Two-way ANOVA with a Duncan *post-hoc* test) and all the experimental groups include RU to induce expression.

Recently, we have demonstrated that the PDF neuropeptide operates during development to determine the fine structure of the dorsal axonal projections of sLNv neurons [33]. As we demonstrated here, Mmp1 affects the circadian remodeling of PDF projections in the adult, concomitantly altering the levels of the neuropeptide. We reasoned that if PDF was responsible for the daily axonal remodeling of sLNvs, rescuing PDF levels in the context of Mmp1 overexpression should reestablish circadian structural plasticity. Indeed, PDF overexpression in the context of Mmp1 overexpression restored circadian structural plasticity of PDF neurons to wild type levels (**Figure 4A**). To directly test a role of the neuropeptide on the plasticity of sLNv neurons, we expressed a specific RNAi to downregulate PDF levels in an adult-specific fashion and analyzed its effect on circadian axonal remodeling. PDF knockdown caused a severe abrogation of the daily remodeling of axonal terminals that rendered the structure in a configuration reminiscent of the one observed in animals overexpressing Mmp1 (**Figure 4B**).

In conclusion, these experiments clearly demonstrate the relevance of the PDF neuropeptide in the daily remodeling of PDF terminals. Moreover, these results led us to propose that daily changes in PDF levels at the dorsal terminals could be responsible for the circadian structural remodeling of the axonal arbor.

### Mmp1 affects neuropeptide-containing dense core vesicles at the dorsal terminals

One particularly intriguing observation made on the course of this work was that Mmp1 deregulation led to altered PDF immunoreactivity. In principle, Mmp1 could be altering PDF levels at the axonal terminals by affecting any step from transcription to neuropeptide processing, release or even degradation either directly or indirectly. To analyze if Mmp1 reduces *pdf* transcription or mRNA stability we measured the steady state levels of *pdf* mRNA by quantitative Real Time PCR (qRT-PCR) in head extracts of control and flies overexpressing Mmp1 or a RNAi against Mmp1 during the early morning (ZT2). No significant differences were observed between control and mutant flies (**Figure S4B**), suggesting that neither *pdf* transcriptional levels nor mRNA stability were grossly affected upon Mmp1 deregulation.

An alternative explanation to the observation that Mmp1 dramatically alters PDF levels at the dorsal protocerebrum is that it could affect neuropeptide release from the dorsal terminals. We tested this hypothesis expressing a GFP fusion to the atrial natriuretic peptide (ANF-GFP) in PDF neurons. When expressed in secretory cells, ANF-GFP was reported to be processed, localized and released in response to physiological signals as an endogenous neuropeptide [36], [37]. Overexpression of Mmp1 reduced ANF-GFP levels, which could be taken as an indication of increased peptide release at all timepoints, suggesting that Mmp1 could promote PDF release from sLNv axonal terminals (**Figure S4C**).

### Recombinant Mmp1 cleaves PDF *in vitro*

**Figure 5:**
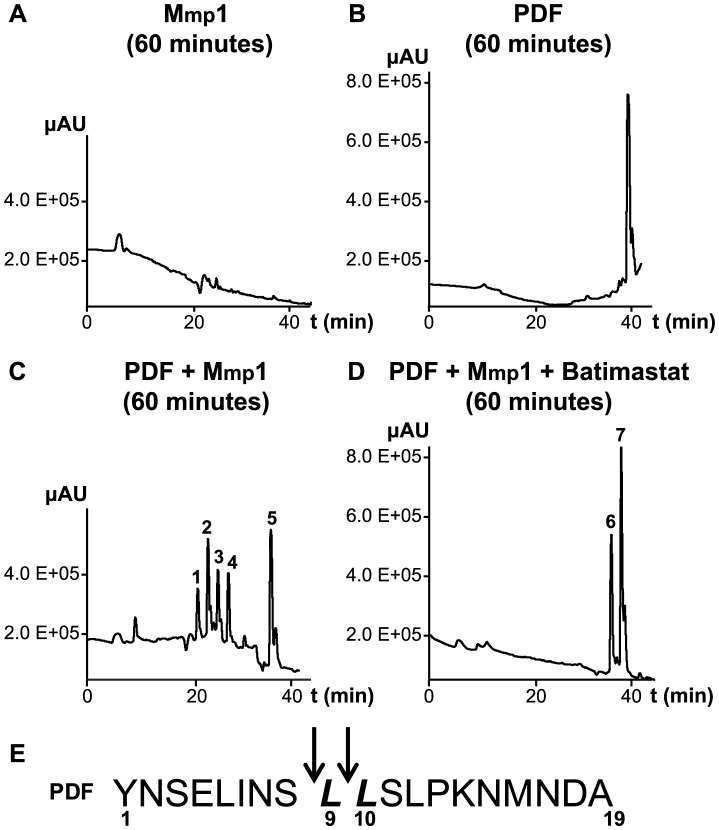
Mmp1 processes the PDF neuropeptide *in vitro*. **A–D.** Reverse-phase HPLC profiles of Mmp1 alone (**A**), PDF alone (**B**), PDF+Mmp1 (**C**) or PDF+Mmp1+Batimastat (**D**) incubated for 1 h at 37°C. **C.** Peaks 1 through 4 contained PDF fragments and the peak 5 was full-length PDF as determined by MS/MS shown in Table 1. **D.** Note the absence of PDF degradation products when Mmp1 was preincubated with the MMP inhibitor Batimastat. Fractions 6 and 7 included PDF 1–19 as identified by MS/MS shown in Table 1. **E.** Schematic representation of Mmp1 preferred cleavage sites within PDF. Arrows indicate the peptide bonds hydrolyzed by Mmp1 as determined by MS/MS analysis. In bold and italics, P1' residues.

To further investigate the ability of Mmp1 to process or degrade PDF, the Mmp1 catalytic domain was expressed in *E. coli* as a His fusion protein. After fast protein liquid chromatography (FPLC) purification and refolding, Mmp1 activity on a previously characterized substrate was confirmed (**Figure S5 A** and [38]). Next, we incubated purified recombinant Mmp1 with PDF for 5 to 60 minutes at 37°C. The reaction products were purified by reverse-phase HPLC [39]. In contrast to recombinant Mmp1 (**Figure 5A**) and PDF alone (**Figure 5B**), co-incubation of PDF with Mmp1 gave rise to four novel peaks consistent with PDF fragments (**Figure 5C** **and Figures S6 A–B**). Moreover, preincubation of Mmp1 with Batimastat, a well-characterized inhibitor of mammalian metalloproteinases [40], prevented PDF cleavage, underscoring that Mmp1 (as opposed to any contaminant potentially present in the original purified fraction) specifically hydrolyzes the neuropeptide (**Figure 5D**). To identify Mmp1 cleavage sites, the four degradation peaks were analyzed by MALDI-TOF-TOF. In the fast eluting fraction, peptides containing the C-terminal sequence of PDF (corresponding to the fragment LSLPKNMNDA of the reported sequence [41]) and to the fragment LLSLPKNMNDA were identified (**Table 1**). Additional fractions included peptides containing the N-terminal PDF sequence (corresponding to amino-acids YNSELINSL), thereby identifying the P1' L-L and P1' L-S as primary sites of Mmp1 cleavage (**Figure 5E**). We also tested whether Mmp2 could degrade PDF *in vitro*. Surprisingly, no novel peaks were detected upon incubation under the same conditions that resulted in Mmp1-directed degradation, even though Mmp2 was able to degrade a previously reported fluorogenic substrate for Mmp2 [42], thus confirming that recombinant Mmp2 displays proteolytic activity (**Figure S5 B–C**).

**Table 1:** MS and MSMS analysis of PDF products detected after incubation with Mmp1.

HPLC Peak^a^	Peptide Fragment	Molecular mass (Da) Obs./Calc.	N-terminal sequence
**1**	10-19	1117.6/1118.55	LSLPKNMNDA+Oxidation
**2**	10-19	1101.6/1101.57	LSLPKNMNDA-amide
		1102/1102.56	LSLPKNMNDA
		1123/1124.55	LSLPKNMNDA+Na
		1117.6/1118.55	LSLPKNMNDA+Oxidation
**3**	9-19	1214.6/1215.64	LLSLPKNMNDA
		1236/1237.64	LLSLPKNMNDA+Na
**4**	1-9	1074.5/1074.53	YNSELINSL+Na
		1090/1090.53	YNSELINSL+Na+Oxidation
**5**	1-19	2136.1/2136.4	YNSELINSLLSLPKNMNDA
**6**	1-19	2151/2136.4	YNSELINSLLSLPKNMNDA+Oxidation
**7**	1-19	2176/2174.4	YNSELINSLLSLPKNMNDA+Na+Oxidation

Obs., observed; Calc., calculated.

a HPLC peaks are depicted in Figure 5.

Taken together these results suggest that Mmp1 could modulate PDF levels at the dorsal terminals, thus contributing to the cyclical changes in PDF immunoreactivity relevant in the control of rhythmic locomotor behavior.

## Discussion

Adult neuronal plasticity is a common mechanism by which neurons adapt their physiology to a changing environment. In particular axonal structural plasticity allows axons to explore new putative postsynaptic targets and, therefore, modify local connectivity as a response to specific stimuli. However, despite its relevance in several neuronal circuits, the molecular mechanisms underlying adult structural plasticity are still poorly understood [18]. In this paper, we studied the molecular mechanisms responsible for the axonal remodeling of sLNvs terminals, a unique type of structural plasticity that comprises spatial long scale changes on a daily basis. We demonstrated a key role of the matrix metalloproteinases and the PDF neuropeptide in the control of circadian structural plasticity of the sLNv axonal terminals. Moreover, we established Mmp1 as a cell-type autonomous regulator of PDF levels, which are key in the transmission of temporal information in the *Drosophila* brain [12], [43]–[46].

### PDF neurons regulate their axonal remodeling autonomously

Throughout this work we extensively showed that deregulation of specific molecules within PDF neurons abrogates circadian structural remodeling of sLNv dorsal terminals, underscoring that PDF neurons can modulate the complexity of the arborization of their own axonal projections. In addition, cell-type specific downregulation and overexpression of Mmp1 or Mmp2 led to increased and reduced axonal complexity reminiscent of the wild type daytime or nighttime configuration, respectively. Interestingly, mRNA steady state levels of both Mmps are enriched in the sLNvs during the beginning of the night [31], which was further confirmed for Mmp1 by immunohistochemistry in the somas of the lLNvs (Figure S1F), suggesting that circadian expression of matrix metalloproteinases within PDF neurons contributes to the daily axonal remodeling.

The fact that pacemaker neurons regulate their own structural plasticity allows this cellular phenomenon to be under tight temporal control. In fact, PDF neurons respond to the neuropeptide PDF [47], [48] and this signal is necessary to coordinate molecular oscillations within sLNvs [11], [49], demonstrating that PDF neurons control diverse aspects of their physiology, in part, cell-autonomously. Extrinsic signals derived from other neurons or even from the glia might add modulation to this autonomous control of structural plasticity.

### Matrix metalloproteinases expressed in PDF neurons control different circadian features

Herein we demonstrate that both Mmps are key players in the control of circadian structural plasticity and their action promotes a reduction in the complexity of axonal arborizations. Matrix metalloproteinases have extensively been implicated in neuronal remodeling during development [26], [27], [50], [51] but, to our knowledge, this is the first evidence of a direct role in adult structural plasticity. Interestingly, minocycline treatment alleviates structural defects in the sLNv axonal terminals of *dfmr1* flies (a fly model of Fragile X syndrome) and this effect appears to be mediated by inhibition of Mmp activity [52]. Although both Mmps are involved in the active remodeling of PDF dorsal arborization, only Mmp2 significantly reduces the total length of axonal terminals. On the other hand, Mmp1 but not Mmp2 significantly reduces PDF levels at the dorsal terminals and in doing so it affects the consolidation of rhythmic locomotor activity. That said we cannot rule out a PDF-independent effect of Mmp1 on locomotor activity. Sequence analysis revealed that *Drosophila* Mmp1 and Mmp2 are more related to different human Mmps than they are to each other [24]; also, Mmp1 seems to be secreted while Mmp2 is retained in the cell membrane [24], [42] therefore different substrates are anticipated for both Mmps. In sum, Mmps modulate relevant aspects of circadian physiology acting at different levels through non-redundant activities.

Mmp1 effect on PDF levels and on the structural remodeling of the dorsal terminals correlates with behavioral arrhythmicity. This observation gives rise to interesting interpretations. On one hand, altering PDF levels or even PDF cycling at the axonal terminals through Mmp1 deregulation leads to arrhythmicity in the locomotor activity paradigm, highlighting once again the relevance of this neuropeptide in the control of circadian behavior [12]. On the other hand, altering Mmp2 expression abolished structural plasticity but did not affect locomotor rhythmicity suggesting that daily axonal remodeling of PDF terminals is not essential for consolidation of rest-activity cycles, in turn opening the attractive possibility that other outputs could depend on such cyclical structural changes [15]. Thus, we propose that pacemaker neurons employ PDF and other classical neurotransmitters to convey time-of-day information to other clock neurons relevant in the control of locomotor activity patterns, and in addition, they communicate via synaptic outputs that are modulated by the daily remodeling of PDF arborizations to regulate other aspects of circadian physiology. In agreement with this possibility, the mammalian suprachiasmatic nucleus uses diffusible signals, like neuropeptides, to daily adjust locomotor activity while depends on synaptic connections to control circadian release of hormones [53]–[55].

### Mmp1 as a regulator of PDF levels at the dorsal sLNv terminals

Mmp1 overexpression leads to a strong reduction of PDF levels in the sLNv axonal terminals while silencing Mmp1 expression clamps PDF levels high, comparable to the daytime configuration. *In vitro* analysis demonstrated that Mmp1 can cleave PDF at specific peptide bonds between the first serine-leucine and between two consecutive leucines; the latter a preferred position for several mammalian MMPs [56]), strongly suggesting that Mmp1 could terminate PDF biological activity. In favor of this possibility, it was reported that similar fragments (PDF1-7 and PDF8-18, targeting the peptide bond between S-L) generated by a different (human neprilysin) peptidase do not activate the PDF receptor [39].

Noteworthy, Mmp1 has been shown to be a direct target of the CLOCK transcription factor [57], enriched in PDF neurons particularly at the beginning of the night ([28], [31], [58] and Figure S1F), which correlates with low PDF immunoreactivity. This time-of-day dependent expression profile, together with the *in vitro* and *in vivo* demonstration of a link between both molecules included here, strongly supports the possibility that endogenous Mmp1 could actively control PDF levels at the dorsal terminals. Interestingly, it has been reported that while most mammalian MMPs are secreted in an inactive form, a few of them contain a RXK/RR motif recognized by furin, which would enable them to be activated by intracellular serin proteinases before they are exported (reviewed in [59]). Furthermore, Mmp1 contains a similar furin consensus sequence (RXKR) that could mediate its intracellular activation [38]. Thus, in principle, MMP1 could be activated within PDF terminals and thus modulate PDF levels at the protocerebrum. Alternatively, MMP1 could degrade PDF in the extracellular space. Lower PDF levels available would give rise to a reduced PDF signaling onto the sLNvs (mediated by PDFR), and in doing so they could alter excitability (Seluzicki et al. 2014) and in turn affect PDF release (Figure S4C and [60]).

### Different signals are coordinated daily to define the pattern of axonal arborizations

Genetic interaction experiments suggest that Mmp1 is involved in the active (and daily) pruning of PDF axonal arborizations through modulation of the activity of EcR and axonal fasciculation; these observations are in line with a recent report showing that MEF2 mediates the activity-dependent remodeling taking place at the PDF dorsal terminals through the regulation of Fasciclin2 [32]. In addition, activation of B1-EcR triggers dendrite remodeling through the action of Mmps during metamorphosis [27]. Interestingly, several proteins induced by EcR (for example, the ABC transporter E23) are enriched at the beginning of the night in PDF neurons [31]. Strikingly, PDF overexpression rescued the decreased axonal complexity triggered by Mmp1 overexpression. Moreover, adult-specific PDF downregulation reduced axonal complexity and rendered the structure in the nighttime configuration, similar to the effect of Mmp1 overexpression. Thus, as it has been reported during development [33], PDF neurons modulate the structure of their own axonal projections via the action of the PDF neuropeptide. Taking these results into account we propose that PDF changes, acting directly via receptors in the sLNvs or indirectly through retrograde signals released by other PDFR immunoreactive neurons [61], could provide relevant feedback information to pacemaker neurons and thereby adjust their connectivity.

**Figure 6:**
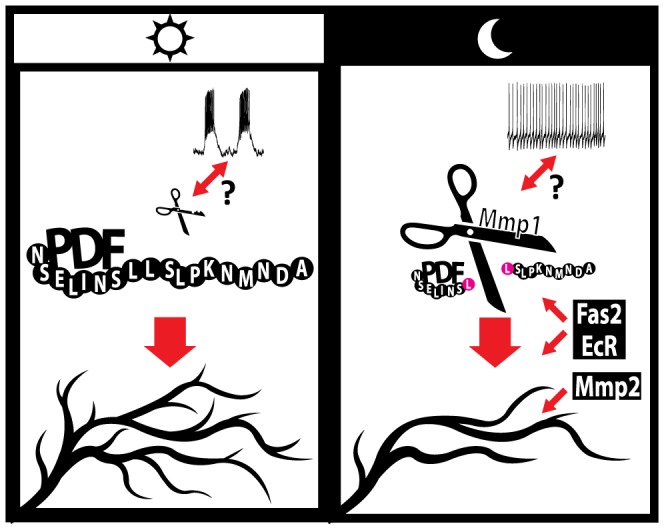
A model for the regulation of circadian axonal remodeling of sLNv neurons. The bidirectional arrow between electrical activity and Mmp1 suggests a possible coordination of both processes. Mmp1 effects on structural plasticity are dependent on the modulation of PDF levels at the sLNv terminals, via direct proteolysis, while Mmp2 appears to act downstream of the neuropeptide. Electrical activity regulates the overall level of complexity but it is not required to determine the circadian aspect of this remodeling. Given our current understanding Fas2 and EcR could act either upstream or downstream of PDF; however, the well-known Fas2 function points to a more direct modulation of circuit structure. Changes in the size of “PDF” and “Mmp1” molecules illustrate oscillations in abundance along the day.

In addition to the role of the molecules identified throughout this work and elsewhere [32], we previously demonstrated that adult-specific electrical silencing of PDF neurons reduces axonal complexity without abolishing circadian oscillations in their complexity, while it clamps PDF levels to the nighttime configuration [30], underscoring that although electrical activity is relevant for structural plasticity, other activity-independent mechanisms underlie axonal remodeling of the sLNv arborizations. During the early morning lLNvs show higher action potential (AP) firing rate compared to the early night [13], [62] and the limited data available on the electrophysiological properties of the sLNv neurons points in the same direction [13]; these changes in electrical properties are accompanied by high and low PDF immunoreactivity in the terminals during day and night respectively [12]. Since activity of a subset of mammalian MMPs can be modulated by electrical stimuli [63], [64], circadian changes in the electrical activity of sLNv neurons could modulate the activity of endogenous Mmps. This modulation would act in concert with the proposed clock- controlled transcriptional regulation of Mmp1 expression [29]. In this context, we propose that during the day, higher sLNv electrical activity along with low Mmp1 levels determine high PDF immunoreactivity in the axonal terminals; peptide signaling in turn promotes a more complex axonal arborization. In contrast, at night, reduced electrical activity and high Mmp1 levels result in decreased PDF immunoreactivity at the axonal terminals and this, along with the action of Mmp2, Fas2 and EcR, reduces the complexity of axonal projections (**Figure 6**).

In sum, the results presented here demonstrate that pacemaker neurons adjust their axonal arbors in a cell-type autonomous manner by recruiting complex mechanisms involving matrix metalloproteinases, modulation of the Ecdysone Receptor, changes in fasciculation and signaling through the PDF neuropeptide.

## Materials and Methods

### Fly rearing and stocks

Flies were grown and maintained at 25°C in vials containing standard cornmeal/agar medium supplemented with yeast under 12∶12 h light∶dark cycles. GeneSwitch expression was induced by transferring 1–4 day old adult males to food containing RU486 (*mifepristone*, Sigma, USA) in 80% ethanol to a final concentration of 200 µg/ml (or 500 µg/ml in the case of UAS-*pdf* rescue experiments) or with the same amount of ethanol (vehicle) in control treatments. All stocks used in this study were described previously: *pdf*-GeneSwitch (*pdf*-GS) was generated in our laboratory [30], UAS-*Mmp1* (chromosomes II and III) and UAS-*Mmp2* (chromosomes II and III) were gently provided by A. Page-McCaw [24], UAS-*Mmp1^RNAi^_B_* and UAS-*Mmp2^RNAi^_B_* by D. Bohmann [65] and UAS-*pdf* by P. Taghert [5]. *w^1118^* (#40015), UAS-*CD8GFP* (#5137), UAS-*CD8RFP* (#27398), UAS-*ANFGFP* (#7001) and UAS-*myrRFP* (#7119) were obtained from the Bloomington Stock Center. UAS-*Mmp1* ^RNAi^ (#101505), UAS-*Mmp2* ^RNAi^ (#107888), UAS-*Dicer2* (#60008 and 60009), UAS-*pdf* ^RNAi^ (#4380), UAS-*EcR* ^RNAi^ (#37059) and UAS-*Fas2* ^RNAi^ (#36351) were obtained from the Vienna RNAi Stock Center. Experiments shown in Figure S1 G were carried out with the RNAi lines generated by the Bohmann laboratory [65].

### Locomotor behavior analysis

Male adult flies (2–4 days old) were placed in glass tubes containing standard food (supplemented with 200 µg/ml RU 486 or vehicle, as indicated in each experiment) and monitored for activity with infrared detectors and a computerized data collection system (TriKinetics, Waltham, MA). Activity was monitored in LD conditions for 4 days, followed by constant darkness for 9–10 more days (DD). Period and rhythmicity in DD were estimated using ClockLab software (Actimetrics, Evanston, IL) as previously described [66].

### Immunohistochemistry and image acquisition

Adult heads were fixed with 4% formaldehyde in 100 mM phosphate buffer pH 7.5 for 30 min at room temperature (RT). Brains were dissected and rinsed three times in PBS with 0.1% Triton X-100 (PT) for 15 min, with the exception of immunohistochemistry against Mmp1 were PBS with 0.6% Triton X-100 was used in all the incubations. Samples were blocked in 7% normal goat serum for 1 h in PT, and incubated with primary antibody at 4°C overnight. The primary antibodies employed were rabbit anti-GFP 1∶500 (Invitrogen, USA), chicken anti-GFP 1∶500 (Upstate, USA), rabbit anti-RFP 1∶500 (Rockland, USA), a cocktail of mouse anti-Mmp1 antibodies 1∶10 (3A6B4, 3B8D12 and 5H7B11 from DSHB) and homemade rat anti-*Drosophila*-PDF 1∶500 [30]. Samples were washed 4×15 min in PT, and incubated with secondary antibody at 1∶250 for 2 h at RT; secondary antibodies were washed 4×15 min in PT and mounted in 80% glycerol in PT. The secondary antibodies used were Cy2-conjugated donkey anti-rabbit, Cy2-conjugated donkey anti-chicken, Cy3-conjugated donkey anti-rat, Cy3-conjugated-donkey anti-rabbit, Cy3-conjugated donkey anti-mouse, Cy5-conjugated donkey anti-mouse IgG1 (Jackson InmunoResearch, USA). Images were taken either on a Zeiss Pascal LSM or a Zeiss LSM 510 Meta Confocal microscope.

### Structural plasticity analysis, PDF and ANF-GFP immunoreactivity

Images were taken with a 40× objective and an optical zoom of 2×. For the analysis of PDF immunoreactivity all pictures were taken employing the same confocal settings and quantification was performed using Image J software (downloaded from http://rsbweb.nih.gov/ij/). Briefly, mCD8GFP signal was adjusted to threshold levels generating a selection that delimit the area of sLNv axonal terminals. This selection was then applied to the PDF channel and mean intensity was measured. A rectangle of the same or a higher area was located outside of PDF neurons and used to subtract background signal. The same protocol was applied to measure GFP levels in ANF-GFP experiments with the exception that mCD8RFP was used to delimit the circuitry. Structural plasticity was analyzed as reported [14]. Total axonal length was measured with the LSM Image Browser Software by following the principal axonal branch of the dorsal projections (illustrated in **Figure S1C**). In all cases the analysis was performed blind.

### Quantitative real-time PCR

Total RNA isolation from fly head extracts was performed using Trizol (Invitrogen, Carlsbad, CA), and FastStart Universal SYBR Green Master (Roche) was used for reverse transcription following the manufacturer's instructions. The real-time assays were conducted in the Stratagene Mx3000P QPCR System (La Jolla, CA) using SYBR green as the detection system and ROX as reference dye. The primers were designed using Primer3 (available online at http://frodo.wi.mit.edu/primer3/). mRNA levels were assessed from four independent RNA extractions and two technical replicates were performed on each sample. Only primer pairs with efficiency between 90% and 110% were used. For *pdf* the following primers were used: Forward ‘GCCACTCTCTGTCGCTATCC’ and Reverse ‘CAGTGGTGGGTCGTCCTAAT’. *RpL49* was used for normalization and the following primers were used: Forward ‘GAACAAGAAGGCCCATCGTA’ and Reverse ‘AGTAACAGGCTTGGCTTGC’.

### Purification and refolding of recombinant Mmps

The catalytic domains of *Drosophila* Mmp1 (735 bp) and Mmp2 (483 bp) were expressed in *E. Coli* as a His fusion protein. Catalytic domains were cloned after PCR amplification using the following plasmids as templates (*Drosophila* Genome Resource Center, RE19818 and SD03462 for Mmp1 and Mmp2, respectively) and then transformed into *E. coli* BL21 AI (Invitrogen). The following primers were used: Forward ‘CAATCGGCACCCGTTTCCACC’ and Reverse ’CTAATACAGTGACTGGATGGCCGC’ for Mmp1 and Forward ‘CAGGGACCCAAGTGGTCCAGAA’ and Reverse ‘AACCTAGTACAACTGCTGAATGCC’ for Mmp2. Expression was induced by addition of 0.2% L-arabinose (Calbiochem), followed by incubation for 2 hours at 37°C. Recombinant Mmp1 was solubilized using 50 mM Tris-HCl (pH 8.5) containing 500 mM NaCl, 2 M urea, 1 mM β-mercaptoethanol, 1 mM PMSF, and 1% Triton x-100. Recombinant Mmp2 was solubilized using 20 mM Tris-HCl, pH 7.6, containing 6 M GdnHCl and 5 mM DTT. Both recombinant proteins were purified by FPLC with a His Trap Ni^2+^-chelating column (GE Healthcare) with a 0–250 mM Imidazol gradient at 0.5 ml/min flow during 50 minutes. After sodium dodecyl sulfate–polyacrylamide gel electrophoresis (SDS-PAGE) analysis, fractions with the recombinant protein were pooled. Refolding of Mmp1 was achieved by dialysis against a 50 mM Tris buffer (pH 7.6) containing 5 mM CaCl_2_, 200 mM NaCL, 50 µM ZnSO4, 0.05% Brij 35, 20% glycerol and 2 mM DTT, O.N at 4°C. Refolding of Mmp2 was obtained by a 2-step dialysis, first against a 50 mM Tris buffer (pH 7.6) containing 5 mM CaCl_2_, 200 mM NaCL, 50 µM ZnSO4, 0.05% Brij 35, 20% glycerol and 2 M GndHCl, for 16 h at 4°C and then against the same buffer containing 2 mM DTT without GndHCl, for 16 h at 4°C. After concentration with a 10 kDa cut-off Amicon Ultra-15 Centrifugal Filter (Millipore), the enzyme preparations were stored with 40% glycerol at −20°C for activity assays.

### Mmp activity assays

The enzymatic activity of purified recombinant Mmps was confirmed using well characterized substrates [38], [42]. Four µg of synthetic fibronectin (Sigma) were incubated alone or together with 250 ng of purified Mmp1, with or without pre-incubation with 1 mM Batimastat inhibitor (Sigma) for 30 min at room temperature. The reaction buffer was 0.1 M Hepes, 0.1 M NaCl (pH 7.4). After incubation for 18 h at 37°C, samples were analyzed by 7.5% SDS–PAGE in Tris–Tricine gels and stained with Coomassie Brillant Blue. The enzymatic activity of purified recombinant Mmp2 was analyzed by using the synthetic OmniMMP™ fluorogenic substrate Mca-Pro-Leu-Gly-Leu-Dpa-Ala-Arg-NH_2_.AcOH (Enzo Life Sciences).

The substrate at 1 µM and Mmp2 at 200 mM were incubated in assay buffer 50 mM HEPES, 10 mM CaCl_2_, 0.05% Brij 35 and 10 µM ZnCl_2_ pH 7.0 for 1 h at 37°C. For its inhibition, Mmp2 was previously incubated with 1 mM Batimastat for 30 min at room temperature. The emission at 393 nm for 1 h and the emission spectra between 350 and 450 nm were measured in a JASCO FP-6500 espectrofluorometer at 37°C (Ex.: 328 nm).

### Peptide synthesis

The PDF peptide was synthesized at NeOmps (France); the primary sequence is YNSELINSLLSLPKNMNDA; since it was originally synthesized for coupling to a carrier during antibody production an additional tyrosine (Y) at position 1 was included. Synthetic PDF was purified by reverse-phase high performance liquid chromatography (HPLC), and evaluated by matrix assisted laser desorption ionization time-of-flight (MALDI-TOF) mass spectrometry (MS) (Cequibiem, Universidad de Buenos Aires, Argentina). The lyophilized peptide was dissolved in 0.1 M Hepes, 0.1 M NaCl (pH 7.4) and aliquoted and stored at −20°C for further use.

### PDF degradation/hydrolysis assay

One hundred and fifty µg of PDF were incubated with 1 µg of purified Mmp1 or Mmp2, with or without pre-incubation (30 minutes at room temperature) with 1 mM Batimastat inhibitor (Sigma) for 5, 15 and 60 minutes at 37°C in 0.1 M Hepes, 0.1 M NaCl (pH 7.4) in a final volume of 100 µl. Reactions were stopped by the addition of 50 µl of 1% (v/v) trifluoroacetic acid (TFA) and the final volume was made up to 500 µl with milli-Q water. The intact/parent peptide and peptide fragments generated by peptidase activity were resolved and quantified by reverse-phase HPLC using a C18 Beckman 5 µm (4.6 mm×25 cm) column and detection at 214 nm [67]. Peptides were eluted with a linear gradient from 0% to 60% acetonitrile in 0.1% TFA at 1 ml/min flow during 1 hour. The differential peaks were analysed by mass spectrometry.

### Mass spectrometry analysis

Molecular masses of intact peptides and the products of Mmp1 degradation were determined (CEQUIBIEM, University of Buenos Aires). Samples were desalted through reversed-phase ZipTip (Millipore, MA) following manufacturer's instructions and analyzed on an Ultraflex II MALDI TOF TOF (Bruker Daltonics) in Reflectron Positive mode and Lift mode using standard instrument settings, and HCCA matrix.

### Statistical analysis

Statistical analyses were performed with the InfoStat package version 2009 (Grupo InfoStat, FCA, Universidad Nacional de Córdoba, Argentina). Normality was tested using Shapiro-Wilks test and the homogeneity of variance was assessed with Levene's test. In all the graphs, experimental groups with different letters indicate statistically significant differences. To illustrate with an example, groups with letters AB are not statistically different from groups coded either with an A or a B but they are statistically different from groups with a letter C. *p*<0.05 was considered statistically significant. For structural plasticity analysis and circadian PDF and ANF-GFP immunoreactivity a two way ANOVA with Circadian Time (CT) and Genotype as factors was performed. In the case of structural plasticity analysis, each independent set of crosses (including one vial per condition) was considered as a blocking factor to reduce the variability between experiments. Locomotor activity was analyzed by a two way ANOVA with Genotype and Treatment (RU or Vehicle) as factors. During two way ANOVA analysis, the significance of the interaction between the two factors (Genotype×CT or Genotype×Treatment) was first analyzed. If the interaction was statistically significantly different (*i.e.*, *p*<0.05) a Duncan multiple comparison test over the Interaction was performed. In the cases where the interaction was not significantly different, we performed the statistical analysis for each factor separately (analysis of principal effects). As a consequence, in those cases, it was not possible to perform all the combinatorial comparisons between experimental groups but only the ones indicated in the corresponding figures. For qRT-PCR analysis differences between genotypes were studied by a one way ANOVA. For the statistical analysis of axonal crosses per ring (**shown in Figure S1**) a repeated measured design was applied where Genotype and CT were considered external factors and Ring an internal factor repeated in space. To simplify the analysis only the axonal crosses with rings, 1, 3 and 6 were taken into account. To analyze ANOVA assumptions, Box's test for homogeneity of variance and covariance matrices, and Mauchly's sphericity test were performed. In all the cases replicates indicate the number of independent experiments. The number of flies used per experiment is indicated in the legend of each particular figure and was adjusted to minimize the internal variance between individuals and considering the specific requirements of the technique.

## Supporting Information

Figure S1 Both Mmps affect the complexity of the axonal arborizations throughout the projections while only Mmp2 promotes reduction of total axonal length. **A.** Overexpression of Mmp1 and Mmp2 with independent transgenic lines triggers similar phenotypes in total axonal crosses. The roman numerals in parenthesis indicate the chromosome of insertion of the UAS line used. Data in Figure 1 corresponds to lines of chromosome II. **B.** Quantitation of axonal crosses between the terminal and the different concentric rings for *CD8GFP*; *pdf*-GS control animals (indicated as “GFP” in the figure) and those overexpressing Mmp1 or Mmp2. Statistical differences were observed between CT2 and CT14 but, for the sake of simplicity, the bars in the graph represent the mean between the two timepoints. The complexity of the axonal arbors is consistently lower in PDF neurons that overexpressed Mmp1 or Mmp2. **C.** Example of the methodology applied to measure the length of sLNv terminals. **D–E.** Quantitation of terminal length after Mmp overexpression (**D**) or knockdown (**E**). No differences between CT2 and CT14 were observed and only Mmp2 significantly affected the total length (Analysis included a two-way ANOVA with a Duncan *post-hoc* test). Deregulation of Mmp2 levels either reduced (upon overexpression) or increased (upon downregulation) the total terminal length. **F.** **Immunostaining in adult brains reveals Mmp1 endogenous expression in lLNvs somas preferentially during the light-dark transition.** **Left panel**. Representative confocal images of *w^1118^* brains stained against PDF (green) and against Mmp1 (magenta). Scale: 10 µm. **Right panel**. Relative frequency of brains with Mmp1^+^ PDF neurons at light-dark transition (ZT22-2) and dark-light transition (ZT10-14). **G.** **An alternative RNAi strain for Mmp1 and Mmp2** [65] **triggers similar phenotypes in structural plasticity.** Representative confocal images of GFP immunoreactivity at the dorsal protocerebrum at CT2 and CT14 on DD4 (**left panel**) and quantitation of total axonal crosses (**right panel**). In all graphs, data represents the average (± standard error of the mean) of independent experiments, a minimum of 15 flies were analyzed per CT/genotype and different letters indicate statistical differences with a p<0.05 (In D–E capital letters indicate differences at CT2, and lowercase indicates differences at CT14). In A and D, “+” in the x axis refers to a single copy of *CD8GFP*; *pdf*-GS while in E and G, *CD8GFP*, *Dcr2*; *pdf*-GS. All the experimental groups with *pdf*-GS include RU treatment to induce expression. Scale: 10 µm.
(EPS)

Figure S2 Dose-dependent Mmp1 effect on the consolidation of rhythmic locomotor activity. Representative actograms (**left panel**) and quantitation of percentage of rhythmicity (**right panel**). Experiments included 2 copies of UAS-*Mmp1*. Genetic and induction controls show robust behavioral rhythmicity while increased Mmp1 overexpression leads to a significant deconsolidation of rhythmic locomotor activity. Data represents average (± standard error of the mean) of 4 independent experiments and a minimum of 55 flies were analyzed per group. Different letters indicate statistical differences with a p<0.05 (One-way ANOVA with a Duncan *post-hoc* test). “+” in the x axis refers to a single copy of the *CD8GFP*; *pdf*-GS transgenes. Endogenous period for each experimental group is indicated in Table S1.
(EPS)

Figure S3 Mmp1 affects axonal remodeling by interacting with Fasciclin 2 and the Ecdysone receptor pathway. **A.** Total axonal crosses from genetic interaction of Mmp1 and Fasciclin 2 (Fas2). Reducing Fas2 in the context of Mmp1 overexpression partially rescues the normal axonal remodeling of PDF neurons. Genotype×CT interaction was not statistically significant and the analysis indicated differences between CT as well as between Genotypes. In the graph different capital letters indicate statistical differences between Genotypes at CT2 while lowercase indicates differences at CT14 (p<0.05 Two-way ANOVA with a Duncan *post-hoc* test). **B.** Genetic interaction of Mmp1 and the Ecdysone receptor (EcR). Downregulation of EcR *per se* fixes PDF neurons in the daytime configuration and, in the context of Mmp1 overexpression, it rescues structural defects. Different letters indicate statistical differences with a p<0.05 (Two-way ANOVA with a Duncan *post-hoc* test). In both graphs. “+” in the x axis refers to a single copy of *CD8GFP*, *Dcr2*; *pdf*-GS, data represents average (± standard error of the mean) of 4 independent experiments and a minimum of 27 flies were analyzed per CT/Genotype.
(EPS)

Figure S4 A. An alternative UAS strain for Mmp1 and Mmp2 triggers similar phenotypes in PDF levels. Different letters indicate statistical differences with a p<0.05 (Two-way ANOVA with a Duncan *post-hoc* test). The roman numerals in parenthesis indicate the chromosome of insertion of the UAS line used. Data in Figure 3 corresponds to lines of chromosome II. **B.** **Mmp1 does not alter** ***pdf*** **steady-state transcript levels.** qRT-PCR analysis of *pdf* mRNA levels from control and misexpressing Mmp1 samples taken at ZT2 (early morning) in LD7. Data represents the average (± standard error of the mean) of 4 independent experiments and *rpl49* was used for normalization. Daytime mRNA levels of *pdf* are not affected by overexpression (with 2 copies of UAS) or downregulation of Mmp1 (One way ANOVA, NS = non significant). In A “+” in the x axis refers to a single copy of *CD8GFP*; *pdf*-GS, while in B, *CD8GFP*, *Dcr2*; *pdf*-GS. **C.** **Mmp1 alters PDF release from axonal terminals.** Analysis of ANF-GFP signal in sLNv axonal terminals. Expression of ANF-GFP was restricted to adult PDF neurons and GFP levels were analyzed on DD4 at CT2 and CT14. Mmp1 overexpression clearly reduced ANF-GFP levels, strongly suggesting a reduction of PDF release. Data represents the average (± standard error of the mean) of 3 independent experiments and a minimum of 21 brains were analyzed per Genotype/CT. Genotype×CT interaction was not significant and the statistical analysis through no differences between CT but significant differences between Genotypes. In the graph different capital letters indicate statistical differences between Genotypes at CT2 while lowercase indicates differences at CT14 (p<0.05 Two-way ANOVA).
(EPS)

Figure S5 Enzymatic activity of recombinant purified Mmp1 and Mmp2. **A.** SDS-PAGE (7.5% acrylamide) stained with Coomassie Blue of Fibronectin alone or incubated with Mmp1 inhibited or not with Batimastat. A control of Mmp1 alone was also included. On the right, mass molecular ladder (kDa). Arrows indicate fragments of Fibronectin after degradation by Mmp1. **B.** Time course of the fluorescence intensity (Int.) emitted at 393 nm by 1 µM OmniPept fluorogenic substrate (red line), 200 nM Mmp2 (green line), Mmp2 inhibited with Batimastat (grey line) and Mmp2 plus OmniMMP fluorogenic substrate (black line). **C.** Emission spectra at 1 h 37°C for the same samples as in B. Note the peak of emission near 400 nm after hydrolysis of OmniMMP by Mmp2 (black line). A.U., arbitrary units.
(EPS)

Figure S6 Mmp1 but not Mmp2 degrades PDF neuropeptide *in vitro*. **A–B.** HPLC of PDF incubated with Mmp1 during 5 and 15 minutes, respectively. Peaks 1–4 indicate PDF degradation products and peak 5 corresponds to full-length PDF, identified by MS/MS as shown in Table 1. **C–D.** HPLC of Mmp2 alone (**C**) or PDF+Mmp2 (**D**) incubated 1 h at 37°C. Note the absence of PDF degradation products.
(EPS)

Table S1 Locomotor activity parameters measured in the behavioral experiments. 1× refers to overexpression with one copy of UAS construct while 2×, with two copies of UAS. In UAS (1×) the roman numerals in parenthesis indicate the chromosome of insertion of the UAS line used. Data in Figure 2 corresponds to lines of chromosome II. * Genotypes include expression of UAS-CD8GFP. # Genotypes include expression of UAS-*CD8GFP*;UAS-*Dcr2*.
(XLS)
